# The Welsh War Hospital Scheme

**Published:** 1914-09-12

**Authors:** 


### The Welsh War Hospital Scheme.

The movement to provide a Welsh hospital for service with the Expeditionary Force, which has been noted in our columns, has met with a splendid response. Those entrusted with the arrangements have completed their work, and up to Saturday last £15,411 had been subscribed towards the estimated cost of £20,000 if the hospital is to be used for six months, though if more money is forthcoming there will be a prolongation of service if necessary. A bed can be named after a donor of £250, and already twenty-nine have been endowed, including one by the Chancellor of the Exchequer.

The hospital is to be a portable one of 100 beds. It will be of galvanised iron, wood, and felt, and will consist of four wards of twenty-five beds each, and of accessory buildings, operating-theatre, kitchen block, *x*-ray, administrative, and pathological block, &c. There will also be a disinfector, laundry, mortuary, refuse destructor, and probably a dairy—if cows are given! It will be erected at Netley, where it will form a section of the big group of hospitals there, and will receive the sick and wounded soldiers coming from the front. Later it may be utilised overseas.

The staff will consist of six medical men and eighteen nurses, also male and female staffs for various other duties. Surgical and pathological work will be a marked feature, and one member will be a qualified dentist as well as a qualified medical man. The commanding officer and senior surgeon of the hospital is Colonel Alfred William Sheen, M.D., who was born at Cardiff on April 30, 1869, and is the son of the late Dr. Alfred Sheen. He was educated at the University College, Cardiff, and went to Guy's Hospital, London. Since his student days Colonel Sheen has been a consulting surgeon at Cardiff in addition to being an honorary consulting surgeon to King Edward VII.'s Hospital, the Cardiff Seamen's Hospital, and the Pontypridd and Bridgend Hospitals. He served in the Boer War for about a year as one of the surgeons to the Imperial Yeomanry Field Hospital. He has been in the local Volunteer and Territorial Forces nearly twenty years, and until recently commanded the 2nd Welsh Field Ambulance of the R.A.M.C., which has left for foreign service. He is the county director of the Voluntary Aid Detachments in Glamorgan organised by the Territorial Association. His ability and experience will prove invaluable. Associated with him in settling the final design of the hospital are Dr. Marcus Paterson, Major Woodley, R.A.M.C., and others.

Messrs. Hall are the honorary architects, and Messrs. Humphreys, of Knightsbridge, the contractors.

### CARDIFF.

The work of equipping the initial number of 520 beds to be used in the 3rd Western General Hospital Section for the sick and wounded soldiers is well advanced. A fair number of soldiers who have met with accidents or have been stricken with illness are receiving attention.

The commanding officer of the 3rd Western General Hospital Section of the Territorial Force is Lieut.-Colonel D. Hepburn, M.D., V.D., and the adjutant and registrar is Major E. J. Maclean, M.D., who is an honorary gynæcologist at King Edward VII.'s Hospital. The principal matron is Miss E. Mont Wilson, matron of that institution. Nineteen medical practitioners and ninety-one nurses comprise the staff for dealing with the sick.

King Edward VII.'s Hospital, Cardiff, has allocated 150 beds, including fifty in the out-patient department, which has been temporarily closed, though casualties and emergency cases are still being treated. Four members of its honorary medical staff and seven of its past and present nurses are on active service, thirteen other sisters and nurses are ready to proceed if called up by the Territorial Force Association, whilst five are available for the Navy. Three of the Cardiff schools have also been utilised. Eighty-six of the beds have been used for ordinary medical and surgical cases, the former type preponderating.

The Red Cross Society is well equipped. Over £1,200 has been subscribed to a Special Equipment Fund, and an attempt is being made to raise the £250 needed to endow a bed in the Welsh Field Hospital. Accommodation for a large number of beds has been offered, but the Society has been requested by Headquarters not to register any at present. It has a hospital at Penarth containing forty beds; one at Cadoxton with twenty beds; and another is now being furnished at Caerphilly for about thirty beds. Some sixty Voluntary Aid Detachments have been registered, and there are four which are unregistered. The president of this section of the Red Cross Society is Lady Plymouth; the honorary organiser is Mr. Lynn Thomas, C.B.; and Colonel J. J. David is honorary secretary.

